# Universal Pharmacare in Canada: A Prescription for Equity in Healthcare

**DOI:** 10.15171/ijhpm.2019.93

**Published:** 2019-10-28

**Authors:** Mohammad Hajizadeh, Sterling Edmonds

**Affiliations:** ^1^School of Health Administration, Dalhousie University, Halifax, NS, Canada.; ^2^Schulich School of Law, Dalhousie University, Halifax, NS, Canada.

**Keywords:** Universal Pharmacare, Health Policy, Equity, Canada

## Abstract

Despite progressive universal drug coverage and pharmaceutical policies found in other countries, Canada remains the only developed nation with a publicly funded healthcare system that does not include universal coverage for prescription drugs. In the absence of a national pharmacare plan, a province may choose to cover a specific sub-population for certain drugs. Although different provinces have individually attempted to extend coverage to certain subpopulations within their jurisdictions, out-of-pocket expenses on drugs and pharmaceutical products (OPEDP) accounts for a large proportion of out-of-pocket health expenses (OPHE) that are catastrophic in nature. Pharmaceutical drug coverage is a major source of public scrutiny among politicians and policy-makers in Canada. In this editorial, we focus on social inequalities in the burden of OPEDP in Canada. Prescription drugs are inconsistently covered under patchworks of public insurance coverage, and this inconsistency represents a major source of inequity of healthcare financing. Residents of certain provinces, rural households and Canadians from poorer households are more likely to be affected by this inequity and suffer disproportionately higher proportions of catastrophic out-of-pocket expenses on drugs and pharmaceutical products (COPEDP). Universal pharmacare would reduce COPEDP and promote a more equitable healthcare system in Canada.

## Introduction

Equity has long been established as a vital component of any nation’s universal health system.^1-6^ Indeed, equity is recommended by the World Health Organization (WHO) as a motivating factor for a given nation’s universal health coverage that also lays the groundwork for any debates on public policy.^2-5^ The WHO^3^ states that equity manifests in healthcare primarily through fair health system financing and fair access to health services. More specifically, equitable healthcare financing includes the use of fair prepayment schemes and protection against catastrophic payments by individual citizens or households.^3,7^ Both equitable healthcare financing and utilization are respectively guided by the universal principles that healthcare should be financed according to ability-to-pay (ATP) and distributed according to need.^8^ Thus, equity and fairness in healthcare has been traditionally viewed, studied, and debated from the two perspectives: equity in healthcare financing and equity in healthcare utilization.

Pharmaceutical or prescription drugs are an essential component of modern medicine. The WHO has stated that every nation should have universal access to essential drugs and medicines.^4^ Additional WHO reports^3,5,6^ outline the importance of having equitable pharmaceutical policies within a more extensive universal health system. Many nations with universal healthcare systems, including virtually every developed country, have enacted universal coverage for medically necessary prescription drugs and medicines. Despite progressive universal drug coverage and pharmaceutical policies found in other countries, Canada remains the only developed nation with universal health coverage that does not include universal coverage for prescription drugs.

Canada’s healthcare system – affectionately known as “Medicare” – is a universal public health insurance system that covers residents for medically necessary hospital and physician services. Coordinated between the federal and provincial governments through the *Canada Health Act* , residents receive first-dollar coverage for necessary hospitalizations, physician appointments, and diagnostic exams regardless of which province they reside in, how old they are, or how much money they have.^9^ Notwithstanding the deep level of coverage for the health services currently covered, universal coverage of drugs has never been implemented in Canada. Provincial governments have individually implemented a variety of public drug coverage programs that alleviate some of the burden on some particular classes of residents, including low-income households, seniors, and those households using social assistance.^2,10,11^ Significant interprovincial variation in drug coverage has led to notable variation in the burden of out-of-pocket expenses on drugs and pharmaceutical products (OPEDP).^11^

Since Medicare was first introduced in Canada by Saskatchewan Premier Tommy Douglas and his social democratic government in 1947,^12^ there has been no shortage of calls to include universal prescription drug coverage for all Canadian residents. Royal Commissions, such as the Hall Commission (1964)^13^ and the Romanow Commission (2002),^14^ as well as numerous recent provincial and federal government reports^2,10,12,15^ have recommended the adoption of universal pharmaceutical drug coverage in some form. Several empirical studies have demonstrated the financial hardships suffered by Canadians because of the uninsured costs of pharmaceutical drugs.^11,16,17^ Even with brief periods of public attention in the past, the topic of pharmacare has remained relatively untouched by Canadian policy-makers because of a lack of electoral incentives and general concern over costs to be incurred by government^18,19^ – despite robust cost proposals by Morgan and colleagues,^20,21^ and Wolfson and Morgan.^22^ Recently, however, pharmacare has become a central topic of public discussion and political discourse, which is demonstrated by its inclusion in the platforms of major political parties for the recent federal election and the establishment of the federal government’s Advisory Council on the Implementation of National Pharmacare.^10^

As Canada looks to implement universal pharmacare over the coming years, it is important for policy-makers to recognize the critical role of equity in the financing and delivery of these new public services. Indeed, the WHO^5^ explains that upon implementing any universal health coverage, nations need to consider the fair distribution of health services, cost-effectiveness, and fair contribution to the health system. In other words, the services should be distributed based on need and financed based on ATP, while being as cost-effective as possible, to achieve the maximum benefit to the target population.^5^ In this editorial, we briefly highlight equity concerns associated with the current pharmacare in Canada. We focus on social inequalities in the financing of drugs and pharmaceutical products in Canada. We provide supporting statistics using pooled data from six nationally representative Survey of Household Spending (SHS) conducted by Statistics Canada between 2010 and 2015 (n = 33 367 households) to empirically demonstrate the equity concerns of the current financing of drugs and pharmaceutical products in Canada. Specifically, we assess provincial, rural-urban, and socioeconomic inequalities in the financing of OPEDP in Canada.

## Provincial Variations in OPEDP

**Figure 1:**
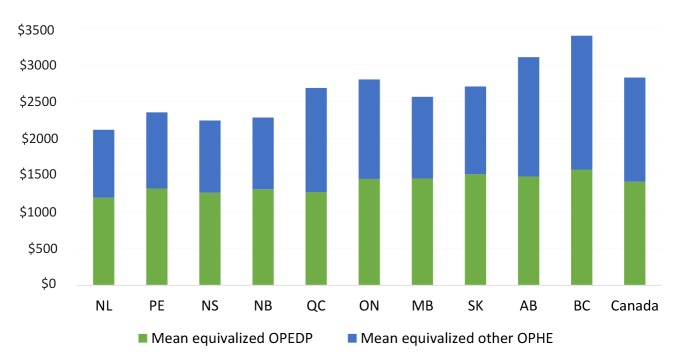
Mean Equivalized Annual OPEDP and Other OPHE Across Canadian Provinces: 2010-2015.
Abbreviations: NL, Newfoundland and Labrador; PE,Prince Edward Island; NS, Nova Scotia; NB, New Brunswick; QC, Quebec; ON, Ontario; MB, Manitoba; SK, Saskatchewan; AB, Alberta; BC, British Columbia; OPHE, out-of-pocket health expenses; OPEDP, out-of-pocket expenses on drugs and pharmaceutical products. Source: Authors’ calculation.

Figure 1 shows the mean annual equivalized OPEDP for the households of each province over the period between 2010 and 2015^[1]^. There exist cross-provincial differences in pharmaceutical coverage, which are demonstrated in the varying levels of mean equivalized OPEDP and proportions of OPEDP to equivalized total household out-of-pocket health expenses (OPHE). As illustrated in the figure, Newfoundland and Labrador had the lowest mean OPEDP in the country, whereas British Columbia had the highest. The proportion of mean equivalized OPEDP to OPHE ranges from 46% (British Columbia) to 57% (New Brunswick and Manitoba).

**Figure 2:**
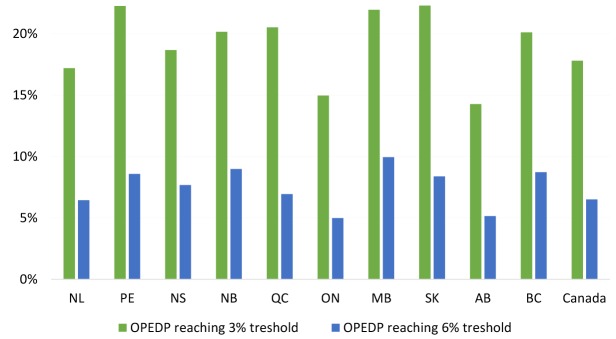
Proportion of Households With COPEDP Across Canadian Provinces Between 2010 and 2015: 3% and 6% of Total Household Consumption.
Abbreviations: NL, Newfoundland and Labrador; PE, Prince Edward Island; NS, Nova Scotia; NB, New Brunswick; QC, Quebec; ON, Ontario; MB, Manitoba; SK, Saskatchewan; AB, Alberta; BC, British Columbia; COPEDP, catastrophic out-of-pocket expenses on drugs and pharmaceutical products; OPEDP, out-of-pocket expenses on drugs and pharmaceutical products. Source: Authors’ calculation.

There are also variations in the number of households facing catastrophic out-of-pocket expenses on drugs and pharmaceutical products (COPEDP) across Canadian provinces (see Figure 2). The percentage of households in a province facing COPEDP of 3% and 6% of total household consumption^[2]^ range from 14% to 22% and 5% to 10%, respectively. The considerable range in households facing COPEDP between provinces over the study period is a result of differences in provincial public health insurance schemes. The absence of universal pharmacare produces variation in drug payments made by the households of different provinces.

## Rural-Urban Differences in OPEDP

**Figure 3:**
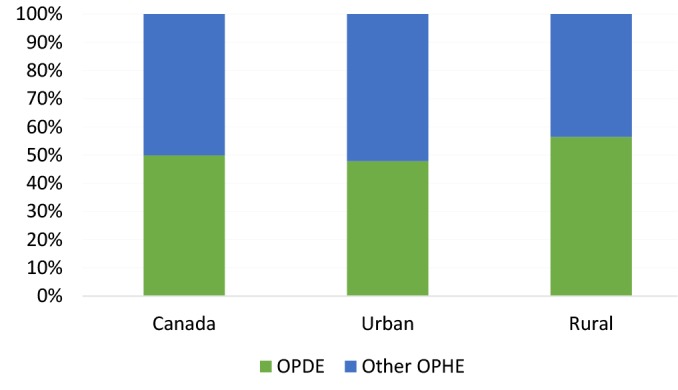
Share of OPDE From Total OPHE in Urban and Rural Areas in Canada: 2010-2015.
Abbreviations: OPDE, out-of-pocket expenses on drugs and pharmaceutical products; OPHE, out-of-pocket health expenses. Source: Authors’ calculation.

**Figure 4:**
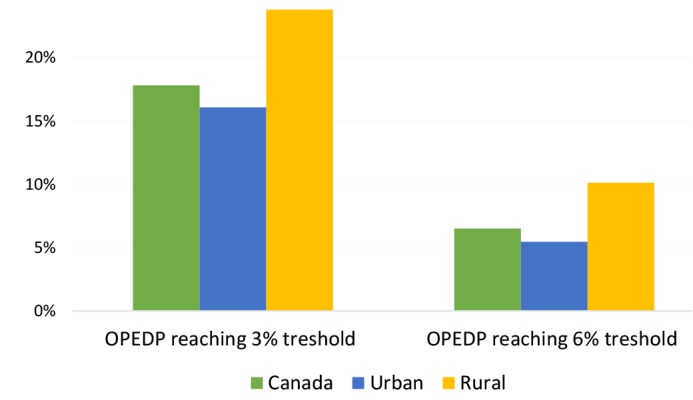
Proportion of Households With COPEDP by Urban and Rural Areas in Canada Between 2010 and 2015: 3% and 6% of Total Household Consumption. Abbreviations: COPEDP, catastrophic out-of-pocket expenses on drugs and pharmaceutical products; OPEDP, out-of-pocket expenses on drugs and pharmaceutical products. Source: Authors’ calculation.

Figure 3 demonstrates the share of drugs and pharmaceutical expenses from total OPHE from 2010-2015 for Canada and urban and rural households ^[3]^. Rural households had a higher proportion of OPEDP from total OPHE (56%) than urban households (48%). Additionally, the percentage of households facing COPEDPwas higher in rural households than urban households for both the 3% (rural area: 24%; urban area:16%) and 6% (rural area: 10%; urban area: 5%) thresholds (see Figure 4). Overall, 18% of Canadian households face COPEDP totaling at least 3% of their total household consumptions. When this threshold is raised to 6%, 7% of Canadians still suffered COPEDP between 2010 and 2015.

## Socioeconomic Differences in OPEDP

**Figure 5:**
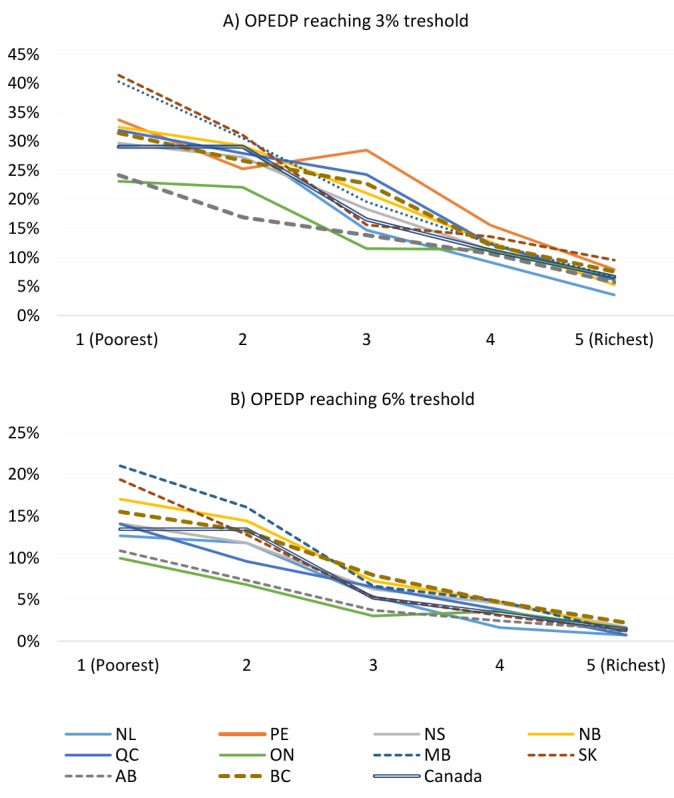
Proportion of Households With COPEDP in 5 SES Quintiles Across Canadian Provinces Between 2010 and 2015: 3% and 6% of Total Household Consumption. Abbreviations: NL, Newfoundland and Labrador; PE,Prince Edward Island; NS, Nova Scotia; NB, New Brunswick; QC, Quebec; ON, Ontario; MB, Manitoba; SK, Saskatchewan; AB, Alberta; BC, British Columbia; COPEDP, catastrophic out-of-pocket expenses on drugs and pharmaceutical products; SES, socioeconomic status; OPEDP, out-of-pocket expenses on drugs and pharmaceutical products. *Note:* Proportions of households with COPEDP in five SES quintiles were not reported for PE in panel B of the figure as per the data disclosure policy of Statistics Canada’s Research Data Centres for small cell sizes (fewer than 5 unweighted households with the COPEDP in one of the SES quartiles).
Source: Authors’ calculation.

Figure 5 features the proportion of households with COPEDP broken down by five socioeconomic status (SES, as measured by equivalized household total consumption) quintiles. Figure 5A uses the catastrophic expense threshold of 3% of total household consumption, and Figure 5B uses the threshold of 6%. Regardless of the threshold, both figures highlight two clear patterns. First, the households with the lowest SES had the highest proportion of COPEDP with progressively lower proportions as the SES quintiles increase. In other words, as household SES increases the proportions of households with COPEDP decrease. The second pattern observed in both figures is the variation of proportions of COPEDP across five SES quintiles. For the lowest SES quintile households, there is substantial variation in the proportion of households facing catastrophic drug expenses across the provinces, whereas the lowest amount of variation is seen in the highest SES quintile. In other words, low SES households suffer from substantial variation in COPEDP across provinces that does not exist for high SES households.

## Concluding Remarks

Equity stands as a long-standing goal of Canada’s healthcare system and is at the heart of progressive pharmaceutical drug policy found across the Canadian provinces.^9,20^ This editorial sought to highlight the equity concerns of the current pharmacare in Canada. Politicians in provincial legislatures across Canada have passed a variety of pharmaceutical drug insurance policies with good intentions, but they have achieved mixed results. Especially in the absence of universal pharmacare, inequality in financial contributions for drugs and pharmaceutical products and levels of COPEDP can be found across the provinces. Moreover, these provincial inequalities are compounded across rural and urban areas as well as different levels of SES; lower levels of SES households were found to have higher proportions of COPEDP.

The considerable differences in provincial policies in Canada have led to people in different provinces receiving different levels of pharmaceutical drug coverage. These variations, in turn, result in substantial inequalities in OPEDP and COPEDP across provinces. Both rural residents and low-SES residents are especially vulnerable to COPEDP. These social inequalities are compounded by other “invisible costs” to society, such as cost-related nonadherence to prescriptions medications.^23,24^ Universal pharmaceutical drug coverage in Canada would reduce system-level inequality and effectively reduce social inequalities in COPEDP.

It is important to note that all types of OPHE are inequitable by their nature.^17,25^ Thus, OPHE other than drugs and pharmaceutical products costs contribute to overall health system inequity should be given their due consideration. As OPEDP constitute a large portion of total OPHE in Canada, the implementation of universal pharmacare would be a large step towards reducing OPHE and promoting a more equitable healthcare system in Canada.

## Acknowledgements

We accessed the SHS at the Statistics Canada’s Atlantic Research Data Centre (ARDC) at Dalhousie University, Halifax, NS, Canada which is part of the Canadian Research Data Centre Network (CRDCN), Hamilton, ON, Canada. We would like to thank the CRDCN for facilitating the access to the SHS and the ARDC analyst Heather Hobson for her support and assistance.

## Ethical issues

We accessed the SHS through Statistics Canada’s Research Data Centre (RDC). Data accessed through the RDC, which follows strict disclosure protocols according to the Statistics Canada Acts, is exempt from the research ethics review board.

## Competing interests

Authors declare that they have no competing interests.

## Authors’ contributions

Both authors contributed to the conception and design of the study. MH performed the analysis and SE helped with the analysis. Both authors contributed to drafting and revising of the editorial.

## Authors’ affiliations

^1^School of Health Administration, Dalhousie University, Halifax, NS, Canada. ^2^Schulich School of Law, Dalhousie University, Halifax, NS, Canada.

## Endnotes


[1] As per the Organization for Economic Co-operation and Development (OECD) publications,^26,27^ households’OPEDP and OPHE were equivalized by dividing them by the square root of household size when we calculated the mean household OPEDP and OPHE.


[2] Catastrophic payments for healthcare are defined as exceeding a certain fraction of household ATP (eg, income or consumptions) in a given period, generally one year. This method aims to demonstrate the disruptive impact of health expenses on households’ living standards because of large healthcare expenditures.^28^ Although budget share (share of healthcare payments from total income/consumption) is used extensively in the current studies, there is no consensus on the threshold of catastrophic payments in the literature. The current studies^11,16,17,25,29-32^ have used the threshold between 3% of budget share to 40% of the capacity to pay (ie, income/consumption minus subsistence expenditure requirements). Similar to the threshold ranges used in the Statistics Canada’s report,^33^ we used 3 and 6% of household consumption as the cut-off.


[3] As per the definition of Statistics Canada,^34^ urban residential regions were defined as population centres with at least 30 000 people.
